# Practitioner reported barriers to parental engagement with Child Health Hubs in North West London disadvantaged neighbourhoods: a COM-B framework analysis

**DOI:** 10.1136/bmjpo-2026-005272

**Published:** 2026-10-22

**Authors:** Jin Qin, Mitch Blair, Dougal Hargreaves, Bina Ram

**Affiliations:** 1 Department of Primary Care and Public Health, Imperial College London, London, UK; 2 Mohn Centre for Children’s Health and Wellbeing, School of Public Health, Imperial College London, London, UK; 3 Division of Psychiatry, Imperial College London, London, UK

**Keywords:** Child Health, Health services research, Health Policy

## Abstract

**Background:**

Families from underserved communities face disproportionate barriers to accessing early years support. In response, Early Years GP Child Health Hub Pilots were set up in three North West London boroughs (Brent, Ealing, Harrow) between 2023 and 2025 to improve access to integrated services, reflecting the NHS 10-Year Plan’s ambition for neighbourhood-based care. The hubs have been funded to continue supporting local communities. However, there is limited understanding of how parents from underserved communities experience these services and the challenges they encounter in engaging with them. This study explored practitioner-reported barriers parents face in accessing and engaging with integrated child health services.

**Methods:**

We used qualitative multiple practitioner-generated case study data specifically for families with children aged 0–5 years from the three pilot sites. We applied the Capability, Opportunity, Motivation-Behaviour (COM-B) framework to analyse the data thematically, guided deductively by the framework and inductively allowing for emergent themes.

**Results:**

Twenty-one case studies, collected between September 2024 and January 2025, were generated by practitioners using information they recorded from contact with each family. Barriers were identified across all COM-B domains and multiple barriers overlapping domains. Capability barriers were reported to be the most common (eg, poor parental mental health, low parenting confidence and limited health literacy). Opportunity barriers included financial hardship, competing priorities, social isolation and family interference. Motivational barriers included fear of judgement, emotional stress and previous negative experiences with services. The case studies also identified several enablers including trusted practitioner relationships, culturally sensitive care, proactive follow-up and multidisciplinary collaboration.

**Conclusion:**

Families living in disadvantaged areas face multiple, intersecting barriers to engaging with integrated services. Child Health Hubs should adopt co-produced, community-informed approaches with parents that integrate health and social support to improve equitable access and sustained engagement for families experiencing adversity.

**WHAT IS ALREADY KNOWN ON THIS TOPIC**

**WHAT THIS STUDY ADDS**

- This study provides learning from novel evidence to inform strategies for neighbourhood-based integrated child health services to better meet the needs of families experiencing disadvantage.

**HOW THIS STUDY MIGHT AFFECT RESEARCH, PRACTICE OR POLICY**

- Findings support the co-design of integrated child health services with families to improve accessibility and acceptability among underserved communities.
- Findings may contribute to informing effective strategies for parent-practitioner relationships and improving system-based approaches to engage and sustain engagement among families experiencing disadvantage.

## Introduction

 Parental engagement is widely recognised as a cornerstone of effective early years health and development services.^1 2^ Rather than being limited to attendance at appointments or programme participation, parental engagement encompasses parents’ active involvement in decision-making, co-producing services and cultivating supportive home environments that complement formal interventions.^1 3^ Previous research indicates that true engagement extends beyond surface-level participation which requires emotional investment, confidence in self-efficacy and readiness for change.^4^ Therefore, effective engagement involves building trusting practitioner-parent relationships, parents believing in their ability to support their child and adopting new strategies to improve outcomes. It is also an evolving service-system process of practitioner-parent collaboration to address parental behavioural elements (eg, adherence to appointments and treatment tasks) and attitudinal elements (eg, belief in the value of interventions).^5^

Parents actively participating in sessions, engaging with practitioners and interacting with their children (high-quality engagement) is associated with enhanced child health outcomes, sustained intervention effects and generalisation of benefits beyond clinical settings (such as language acquisition, school readiness and social-emotional well-being).^6–8^ Given these benefits, parents function as critical partners and gatekeepers in access and sustained engagement with services; willingness and ability to engage, directly influence the uptake and effectiveness of child health integrated services.^9 10^ Engagement with services has been shown to be facilitated by parents’ perceived benefits, content that is practical and culturally appropriate, flexible delivery, supportive environments and responsive facilitator practitioner practices.^9^ Ensuring high-quality parental engagement is crucial to the local child health integrated services and also to broader NHS Integrated Care Systems and local Integrated Care Boards (ICB), which rely on family-centred participation for coordinated and continuous care.^11^

Co-production has emerged as a key approach for translating parental engagement into child health service practice.^12^ This approach results in interventions that are more responsive, acceptable and sustainable, ultimately improving the quality of care,^1^ and reinforcing national and international policy priorities that position parents as active partners in the design and delivery of integrated child health services.^12^ Frameworks such as the NHS Long Term Plan,^13^ Best Start for Life Vision,^14^ the WHO Framework on Integrated People-Centred Health Services^15^ and the Nurturing Care Framework^3^ increasingly emphasise co-production, family-centred care and community engagement as fundamental to improving service accessibility, responsiveness and equity. However, despite these initiatives, there is little evidence of consistent, system-wide structures that fully embed parental input in service design and delivery.^16^

Previous research has demonstrated the utility of the Capability, Opportunity and Motivation Behavioural (COM-B) framework for understanding parental engagement with child-focused interventions and support services.^17–19^ However, limited research has applied COM-B to explore how parents engage with integrated child health services, particularly those experiencing socioeconomic disadvantage.

In North West London (NWL), the Early Years GP Child Health Hubs Pilots integrated programmes (Optivita in Harrow, Create in Brent and StartWell in Ealing) were developed to reach underserved families with children aged 0–5 years.^20^ Despite policy emphasis, in NWL there is no peer-reviewed research exploring parental engagement on integrated child health services focusing on underserved families. This highlights an important gap given the structural, cultural and psychosocial factors that may constrain reach.

The aim of this study is to use practitioner-reported case-study data, collected as part of the NWL Early Years GP Child Health Hubs Pilots evaluation,^21^ to understand the perceived barriers that underserved communities experience when engaging with Child Health Hubs in NWL.

## Methods

Our study uses a qualitative multiple case study design. Case studies were collected for the NWL Early Years GP Child Health Hubs Pilots evaluation^21^ between September 2024 and January 2025. These were compiled by early years practitioners working with each early years pilot site. Data of interest were families with children aged 0–5 years. During these early years, foundational behavioural, cognitive and physical patterns are established. Intervening early prevents exacerbating poor health, mitigating developmental risks before they become difficult to address in adulthood.^22^

### Case study data

Case study data were captured using a template (online supplemental material 1) designed by the Optivita (Harrow) Hyper Local Preventative Care Team (HLPCT). The template included specific questions and required minimal guidance to complete. HLPCTs at each pilot site identified families to include as case studies. The data comprised practitioner-generated accounts of families’ experiences, challenges, support received and perceptions of Child Health Hub services. The templates were completed retrospectively; practitioners referred to their recorded notes during patient contact (in-person home visits/meetings/events, phone calls, emails, text messages). The final section of the template requested practitioners’ own reflections and learning from each case. Practitioners’ who compiled the case studies were community workers (Social Prescribing Link Workers, Community Health and Wellbeing Workers, Family Link Workers (FLWs)), respiratory clinic practitioners and those facilitating group-based activities (eg, Children’s Centres).

### Analysis

We descriptively describe the number of case studies per site, and how families came into contact with GP Child Health Hubs. The COM-B framework^23^ was integrated deductively within an interpretivist approach^24^ to provide a systematic means of categorising the behavioural determinants of practitioner accounts of parental engagement. Inductive approaches were used to identify determinants to ensure the analysis remained responsive to factors emerging from practitioners’ accounts that were not anticipated by the COM-B framework.^23^

Case studies were read independently by two researchers (JQ, BR), and sub-codes were refined through comparison across cases to ensure consistent application. NVivo (v15) software was used to map emerging data systematically to the COM-B domains and sub-domains.^25^ Coding continued until no new themes were identified and thematic saturation was reached.^26^ The analysis synthesised practitioner accounts of parental experiences at a thematic level, focusing on identifying recurring patterns rather than illustrative verbatim extracts.

### Ethics

This study used data obtained for the evaluation of the Early Years GP Child Health Hub Pilots,^21^ considered a service evaluation by Imperial College Research Ethics Committee, therefore, formal ethical approval was not required. Data was provided by NWL ICB which operated under its own governance framework. We followed all UK General Data Protection Regulation principles to support accountable practices in data use, storage and sharing. Only case studies where practitioners had obtained permission from families were shared with the evaluation team.

### Patient and public involvement

Patient and public involvement was undertaken for the wider evaluation, including a series of workshops and public events designed to inform local communities of the early years integrated services available, and to ascertain health and care priorities in the local areas.^21^ Contact with families during these events was included, if applicable, in the practitioner-generated case study reports.

## Results

### Sample characteristics

A total of 21 case studies were provided by the pilot sites. Harrow provided the highest number of case studies, n=15 (71.4%), Ealing provided 5 (23.8%) and Brent provided 1 (4.8%). Differences in the number of case studies provided by each site, specifically the limited number from Ealing and Brent compared with Harrow, reflected delays to pilot start dates and limited resources^21^ to complete case study templates.

### Referrals

A total of 11 (52.4%) families were referred to the GP Child Health Hub through their GPs, 2 (9.5%) from a parental support group session, 1 (4.8%) through attending a Children’s Centre and 4 (19.0%) were referred from an early years practitioner (referral data for 3 (14.3%) participants were missing). 13 case studies were with mothers only and 8 with both parents.

### Perceived barriers to parental engagement

Psychological capability was consistently reported by practitioners as barriers to parental engagement (14 case studies), followed by social opportunity (13 case studies), while motivation factors were less frequently documented (summary presented in table 1; case study details presented in online supplemental material 2).

**Table 1:** Practitioner-reported perceived barriers to parental engagement with child health hubs across COM-B domains

COM-B domain	COM-B sub domain	Barriers	n=21
Capability	Physical capability	Language barrier	7
		Caregiving capacity	1
	Psychological capability	Mental health challenges	14
		Parenting confidence and self-efficacy	9
		Service literacy	3
		Health literacy	1
Opportunity	Physical opportunity	Financial constraint	9
		Competing priorities	8
		Housing insecurity	7
	Social opportunity	Social isolation	13
		Family interference	2
Motivation	Reflective motivation	Fear of judgement	5
		Previous negative experiences	2
	Automatic motivation	Emotional stress	7

### Capability

#### Physical

Language barriers were the most common physical capability issue, identified in 7/21 case studies. Practitioners reported that parents with limited English struggled to understand service information, communicate with health professionals and complete routine forms. This was particularly evident among families who had recently migrated to the UK; in one case study, the practitioner reported that a mother required an interpreter to participate in maternity and early years activities, while in another case study, the SPLW described the mother as ‘very difficult to engage’ because language barriers contributed to missed baby health check appointments and attendance at parent group sessions. In another case, the practitioner reported that a mother’s recovery from illness reduced her capacity to attend health services; her physical health challenges were compounded by recent migration, limited spoken English, marital breakdown and limited family support.

#### Psychological

Psychological capability barriers, primarily poor parental mental health, were the most prominent finding, reported in 14/21 case studies. Practitioners reported that anxiety, depression and chronic stress reduced parents’ confidence, motivation and ability to engage with health services. For example, one mother with perinatal mental health difficulties was noted to repeatedly decline playgroup invitations despite sustained support from FLWs, while another who was experiencing severe anxiety and depression was unable to leave her home and consistently declined referrals and follow-up. Low parenting confidence and self-efficacy were identified in nine cases, particularly among first-time mothers, and for some were exacerbated by breastfeeding difficulties and criticism from extended family.

Difficulties navigating health, education and welfare systems were identified as barriers in three case studies. Parents in these case studies relied heavily on family members to access services and benefits, often unaware that Child Health Hubs could provide practical support. One family was able to access services but struggled to understand complex treatment information for their children who had multiple health conditions.

### Opportunities

#### Physical

Financial constraints were identified in nine of the 21 case studies. Families were reported to be struggling to meet essential costs including rent, food, childcare and other expenses (eg, purchasing pushchair). In one case study, financial hardship was exacerbated by the cost of specialist equipment for twins with autism spectrum disorder (ASD), while another mother was reported to be anxious about benefit entitlement and financial insecurity during her maternity leave. Housing insecurity was reported in seven cases and included poor housing conditions, overcrowding and the threat of eviction. These conditions directly affected children’s health; for example, damp and mould contributed to one child’s hospital admission, while children in another family were all experiencing respiratory conditions. Overcrowding also reduced parents’ autonomy, with practitioners reporting that interference from extended family was affecting mothers’ decisions about their child’s health and care.

Competing priorities were identified in eight case studies. This included employment, caring for multiple children often with additional needs, supporting extended family, parents managing their own health and addressing financial and housing difficulties. One single mother cared for three young children, including one with ASD and another with severe eczema requiring intensive care, while another was described with persistent exhaustion while raising six children. One mother was reported to express concern that returning to work after maternity leave would limit her engagement with Child Health Hub services.

#### Social

Social opportunity barriers, particularly social isolation and limited support networks, were the most frequently reported opportunity-related challenges (13/21 case studies). Families described a lack of nearby family and friends, strained relationships, limited partner support and difficulties establishing new networks. The absence of informal support reduced access to childcare, practical advice and emotional encouragement. In two cases, interference from extended family further undermined parental confidence and autonomy, discouraging engagement with Child Health Hub activities.

### Motivation

#### Reflective

Reflective motivation barriers (beliefs and decisions that influence behaviour) centred on parents’ fears of being judged, limited trust in professionals and beliefs that services would not respond to their needs. In five of the 21 case studies, practitioners described parents’ concerns about being criticised for their parenting or personal circumstances. This led parents to either avoid engagement with services, withhold personal information or decline support. In one case, anticipated judgement prevented a mother from expressing her needs, limiting practitioners’ ability to provide tailored support. Previous negative experiences with health services reinforced these beliefs. One mother was reported to mistrust practitioners after a health visitor failed to follow-up concerns she had disclosed, while another was reported to disengage after feeling unsupported by healthcare professionals following her child’s prolonged hospitalisation. One mother was also reported to avoid community settings following previous trauma; despite this avoidance being informed by her past experiences and expectations of services, it also reflected an emotional response to trauma, illustrating the overlap between reflective and automatic motivational processes.

#### Automatic

Automatic motivation barriers (emotional responses and habitual reactions that shape engagement) were identified in seven of the practitioner-generated case studies; parents describing emotional responses that reduced their capacity to engage with services. Parents were commonly reported to feel overwhelmed, anxious or unable to cope with everyday demands. Emotional stress was caused by many different factors including bereavement, relationship breakdown, family conflict and competing responsibilities. One mother was reported to be grieving the death of her own mother while coping with separation from her partner, which was impacting her ability to breastfeed her child. Emotional distress, reported by practitioners across several case studies, was a common barrier limiting parents’ capacity to engage consistently with Child Health Hub services.

### Enablers for engaging with Child Health Hubs

Despite the practitioner perceived parental barriers, several enablers were also reported that supported families’ engagement with integrated care services (online supplemental material 3). Central to successful engagement was the development of trusting relationships with practitioners. Practitioners reflected that families responded positively when professionals provided consistent, non-judgemental support, active listening, reassurance and safe spaces to discuss concerns. Sustained relationship-based approaches were particularly important for parents experiencing social isolation, low confidence, mental health difficulties or previous experiences of distrust of services, recognising that engagement often required time and repeated contact.

Multi-disciplinary collaboration and coordinated support across health, social care, education, housing and voluntary sector services helped address interconnected practical, social and emotional needs. Addressing practical barriers, including housing instability, financial hardship, language needs, childcare and relocation challenges, alongside emotional support, improved parents’ confidence, reduced isolation and anxiety, and increased their ability to seek help when needed. The case studies also highlighted the importance of ongoing monitoring and flexible support; families’ needs frequently changed over time and some issues could escalate without continued engagement. While some families were reported to decline or struggle to engage with available services, important facilitators of early intervention sustained engagement included practitioners to provide culturally sensitive practice and holistic, family-centred care, particularly for families of children with additional needs.

## Discussion

We found barriers to parental engagement, as perceived by practitioners, across all COM-B domains in our analysis of 21 case-studies. The most frequently reported occurring barrier was psychological capability, followed by physical opportunities. Physical capability, social opportunity and motivational barriers were less commonly documented but nevertheless contributed to perceived barriers parents’ experienced when engaging with child health hubs and associated activities. Importantly, in all of the case studies, families were reported to be experiencing multiple, interdependent barriers across more than one COM-B domain, suggesting that difficulties with engagement may be cumulative rather than isolated. Despite these challenges, we also identified enabling factors from practitioner accounts which encouraged families’ engagement with services.

In our study, we found that the COM-B components were interrelated; barriers in one domain often exacerbated those in another. This interdependence reflects a core COM-B principle, that parents need sufficient and concurrent capability, opportunity and motivation for engagement to occur.^23^ Insufficiency in any one component can undermine engagement, despite others being addressed.^23^

The strongest theme to emerge from our study was the role of psychological capability. Parents living with depression, anxiety or chronic stress were reported by practitioners as most likely to withdraw from services despite practitioner offers of support. This aligns with prior evidence that parental mental illness reduces responsiveness to children’s needs and can disrupt relationships with health professionals.^27–29^ In COM-B terms, poor mental health reduces the psychological resources needed to process information, plan actions and follow through on advice. Findings of low parenting confidence, especially among first-time mothers, support Staudt’s model of engagement,^5^ which highlights that genuine participation requires behavioural adherence and attitudinal confidence. Our findings support those from international studies that have found associations between reduced parental self-efficacy and poor uptake of preventative child health services.^4 30 31^ In practice, low confidence translated into hesitancy; parents were reported to doubt their ability to benefit from sessions and avoided group activities altogether. Confidence was not only a practitioner reported barrier to initial access, but also to sustained participation in activities. A further challenge to capability was service and health literacy. Practitioners commonly identified that parents were unfamiliar with navigating the welfare system or struggled to follow treatment plans for their children. Similar patterns have been observed in Australia, with low health literacy linked to distrust of services and reduced uptake of integrated care models.^30^ This demonstrates that capability is not solely about psychological resilience, but also about understanding information. Although less frequently reported, physical capability barriers relating to language emerged as a constraint on engagement. Without adequate interpreter provision, parents struggled to complete forms, access information and communicate effectively with practitioners. This finding reflects wider evidence that insufficient translation support systematically excludes some groups (eg, asylum-seeking and migrant families) from healthcare.^32 33^

Opportunity-related barriers, particularly in the physical domain, identified that parents lived with financial insecurity; poor housing conditions, overcrowding and threats of eviction. Parents with multiple children or additional caregiving responsibilities were also reported by practitioners of feeling overwhelmed by competing demands on their time. These issues often intersect, placing families in a state of ‘crisis management’, where stressors consistently take precedence over longer-term child developmental or health goals. Previous research has also demonstrated that financial stress,^30 34 35^ housing insecurity^36 37^ and competing responsibilities such as employment, caring for other children or supporting family^35^ all contribute to reduced parental engagement in childcare-seeking behaviours.

Social isolation, particularly for single parents and among newly arrived migrants who had no extended family or local support networks, emerged as a perceived challenge by practitioners. Isolation reduced access to informal childcare, peer encouragement and everyday parenting advice, all of which facilitate engagement with integrated services.^38 39^ In our study, isolation was frequently reported to be coexisting with other difficulties such as poor parental mental health or housing insecurity. Social opportunity can be constrained by the absence of supportive networks and the presence of unsupportive relationships. Within COM-B, such interference restricts parents’ ability to be exposed to opportunities of constructive social influences.

We found motivation-related barriers to be the least documented among our case-studies, but nevertheless, they played an important role in shaping parents’ willingness to engage with Child Health Hubs as perceived by practitioners. In practice, this meant that while some families maintained contact with practitioners, they withheld crucial information about their circumstances. In turn, practitioners reported that this limited their ability to provide tailored support. Our findings add to the literature; effective engagement requires affective investment and openness,^4^ however, investment is diminished when parents anticipate judgement and criticism rather than support. Comparable evidence from a study in Australia^31^ showed that a trusting relationship between parents and practitioners emerged as a key motivator for parents sustaining engagement with integrated hubs.^31^ Furthermore, practitioners reported that parents experiencing emotional stress affected their ability to engage with services despite expressing a desire for support, and practitioners’ persistence. In COM-B terms, emotional stress disrupted automatic processes that normally influence and direct behaviour, creating a feedback loop where reduced engagement further exacerbated feelings of isolation and inadequacy.^23^

The enablers identified from our case studies align with international evidence, demonstrating that relational trust and culturally safe practice are central to the success of integrated early years hubs in Australia.^30 37^ Importantly, they highlight that Child Health Hubs can function not only as access points for service provision, but also as protective relational spaces, where parents feel listened to, supported, respected and understood.

### Strengths and limitations

Our study has several strengths. The case studies offered a rich, context-specific narrative of parent-service interactions within a real-world service delivery setting, contributing to its novelty. Using data collected through a service evaluation of GP Child Health Hub pilots in NWL provided a unique opportunity to address parental experiences of engagement with early years’ services among underserved families. Another key strength is our application of the COM-B framework to systematically categorise barriers. This ensured that our analysis considered a full spectrum of influences and did not focus on a single dimension. To the best of our knowledge, no previous UK research has examined parental engagement with integrated child health services through a behavioural perspective in NWL. This theory-driven approach enhances interpretive depth and aligns with the Behaviour Change Wheel model, strengthening the translation of findings into policy-relevant implications. However, several limitations must be acknowledged. We did not have access to detail describing how HLPCTs identified families to include for the case studies and how these were selected for sharing with the evaluation team. Parents’ experiences were generated by practitioners’, with no parental oversight into the interpretation of the findings. Consequently, some parental experiences may have been obscured, misinterpreted or underrepresented.^40^ A few identified barriers, for example, language difficulties, could reasonably be classified within two COM-B domains: capability (understanding English) or opportunity (lack of language-appropriate provision). The dataset, despite providing valuable cross-sectional information, lacked longitudinal detail. We were unable to determine whether identified barriers were transient or persistent, or to assess how engagement evolved in response to changing family circumstances or sustained practitioner involvement. Finally, the distribution of case studies was heavily skewed towards one borough. This imbalance restricts representativeness across NWL boroughs; each has its own distinct demographics, service configurations, and socioeconomic profiles. Furthermore the modest sample size (n=21) constrains generalisability both within NWL and to the wider national context. Despite these limitations, our study benefits from novelty and a robust theoretical and methodological framework.

### Cumulative and interdependent barriers to engagement

Our study provides a novel contribution. The identification of cumulative and interdependent barriers has been under-recognised, or assessed as isolated issues in previous UK studies.^32 35 36^ Our study demonstrates that families may rarely experience a single barrier in isolation, illustrating how barriers reinforce one another, pushing families into a cycle of disengagement. By applying COM-B in this context, our study highlights how structural, cultural and psychosocial determinants interact to constrain parental behaviour, offering a more comprehensive account of parental engagement as perceived by practitioners. Previous evaluations of Child Health Hubs have focused predominantly on system-level outcomes (hospital utilisation, referral pathways, cost-effectiveness).^41–44^ When parents’ engagement has been examined, evidence has largely been limited to quantitative satisfaction measures,^41 45^ with only one UK qualitative study exploring family experiences within a different integrated early years model.^37^ Although qualitative evidence from Australian Child and Family Hubs provides useful insights,^30 31^ further research is needed to understand how integrated child health services can best engage diverse families, evaluate implementation across different contexts, and identify strategies that reduce inequalities in access and sustained engagement. Recognising the cumulative impact of barriers is essential. Solutions that target a single issue in isolation are unlikely to succeed. Instead, a comprehensive, multi-level approach is required to lower the overall burden of disadvantage faced by marginalised families and to improve Child Health Hub accessibility and responsiveness.

### Implications for policy

Our findings have important implications for the design and implementation of integrated child health services. Interpreter services, translated materials, bilingual staff and workforce training in cultural competence and parental mental health are essential to improve equitable access among underserved families, including practitioners to receive trauma-informed and anti-stigma training, alongside the involvement of community connectors and peer advocates with lived experience to build trust and encourage service use. Child Health Hubs should also move beyond signposting towards genuine service integration; as barriers are interconnected, there is greater risk of disengagement. Involvement of parents, particularly those from underserved communities, should underpin the design, delivery and evaluation of Child Health Hubs.

Our findings may also suggest a need to move beyond accounts of engagement that locate barriers primarily within parents or their circumstances. Although understanding parental experiences and priorities may directly influence their engagement with Child Health Hubs, their experiences may also be influenced by the healthcare structures and services with which they interact. Applying the COM-B framework provides an improved understanding of these influences, particularly through the domain of opportunity, which includes the social and environmental conditions that may enable or constrain behaviour, and may be created or reinforced through service structures, professional practices and power imbalances. For example, practitioners’ communication with parents, assumptions about parental needs, and fragmented services may influence parents’ social and physical opportunities to engage, while power imbalances may influence what is understood as meaningful engagement. Future research should consider COM-B alongside a more critical examination of the service context. This would help to ensure that lack of engagement is not placed predominantly on parents, and instead recognise engagement as a relational process shaped by both parental circumstances and the systems in which services are delivered.

National standards, supported by robust monitoring from ICBs are needed to ensure equitable provision and inform continuous improvement. Together, these measures would help create more accessible, responsive and equitable Child Health Hubs, while advancing the ambitions of the NHS Long Term Plan and the Best Start for Life agenda.^13 14^

## Conclusion

As reported by practitioners, families living in disadvantaged areas of NWL face multiple, intersecting barriers to access integrated child health services. To achieve equitable access, Child Health Hubs should provide integrated support addressing health and social needs together, rather than through fragmented interventions. Proactive approaches to reducing barriers could include flexible and accessible provision, community-based outreach, sustained relationships with families and building trust with local organisations to ensure services are accessible, acceptable and responsive. However, longer-term evaluation is needed to understand how parental engagement evolves over time, particularly how participation can be sustained among families experiencing adversity.

## Supplementary material

10.1136/bmjpo-2026-005272 online supplemental file 1

10.1136/bmjpo-2026-005272 online supplemental file 2

10.1136/bmjpo-2026-005272 online supplemental file 3

## Data Availability

No data are available.
