# Supplementary material for: Practitioner reported barriers to parental engagement with Child Health Hubs in North West London disadvantaged neighbourhoods: a COM-B framework analysis
Source: BMJ Paediatr Open. 2026 Oct 22;10(1):e005272. doi: 10.1136/bmjpo-2026-005272 (PMC13691901; doi:10.1136/bmjpo-2026-005272)
Supplement: Supplementary file 1 [file bmjpo-10-1-s001.pdf]

Supplementary Material 1. Case study template

SITE: [redacted]

**Case Study**	[redacted]
**Area of work**	**SPLW and CHWW**
**Number of visits/sessions:**	2 sessions, plus numerous emails and phone calls.
**Completed by:**	[redacted]
**Consent received:** If identifiable Case Study	YES

**Family Description**
Mum lives at home with 2 boys under 4. Dad visits, but they are separated. There is a history of them not getting on, and a Section 17 Safeguarding was in place.
**How was contact Initiated**
This referral was made by the GP.<br><br>Referred with the following needs:<br><br>• Help with finances- e.g. can't afford rent<br>• Housing- facing eviction<br>• Mental Health- severe anxiety<br>• Housing problem- damp and mould
**Relationship building**
In my role as SPLW I first met the parent at a group at [redacted] by chance, then arranged to meet her at [redacted] mid Oct 2024 for a face to face one to one.<br><br>I have maintained contact with mum through phone calls, messages, email and face to face meetings. A CHWW colleague followed up when I was on leave.
**Key Issues**
At first meeting, issue that arose that were discussed:<br><br>• Relationship- Mum discussed situation at home- partner lives in [redacted] but sees the children a lot. She describes him as 'a good father'. She has decided she can't live with partner, as she queries if he has OCD and he constantly criticises her for the state of the house etc. He doesn't feel there is anything wrong with him.<br>• Housing- she needs an up-to-date tenancy agreement. The landlord substitute, who mum deals with, won't take her to court, but she is struggling with the rent.<br>• Discussed possibility of Domestic Abuse charities-she is aware. She has been under IAPT for her mental health and will contact them again if needs more.<br>• Children- she attends a [redacted] nearby, and [redacted] playgroup. She is concerned about her 2 year old's speech and language development. His nursery apparently said they would focus on it when he started there, but they haven't fed back to her on it. She is going to ask them if they have done this then, if they have, get the nursery to refer him to SALT service with concerns. SPLW to liaise with SALT.<br>Later discussions revealed concerns regarding children's health due to the mould and damp in their home. One son has had admission to A and E regarding breathing and now has inhalers.

**Support provided?**
How have you supported this person/people?<br>What ongoing support is being offered?<br>Brief outline of emotional and practical support.<br>What type of referrals were made, and how was partnership working maximised?<br><br>Organisations parent signposted to:<br>• [redacted] Centre- housing advice<br>• [redacted] - Warm Hubs<br>• Healthy Homes<br>• [redacted] Baby Bank<br>• Speech and Language Therapy<br>• Talk and Play sessions at the Children's Centre<br>• Talking Therapies- she has been there in the past and will self - refer again if she feels the need<br>Also suggested:<br>• Asking GP for supporting letter re the children's health to the council to support housing issues?<br>• Discussed DV organisations with her- she is aware<br>I have also:<br>• Written a letter to Environmental Health on her behalf copying in the local councillor<br>• Given her some advice on ways to progress the older boy's language skills<br>• Liaised with SALT to find out for mum that the nursery need to work on the older child's speech and language for a period then refer him to SALT if concerned. The nursery can fill out the referral form, mum can do a self-referral or they can do one together.<br>• Regularly checked in with mum by phone/message/email
**Outcomes**
General<br>• Mum now has knowledge of resources to help her get support for her needs.<br>• SPLW has provided her with time and safe space to air her concerns
**Community Engagement**
Mum goes to groups at the children's centres and attends [redacted] playgroup.
**Parent/Family Comments**

Please share any relevant photos to accompany the case study here:
Do you have permission from the resident to use these photo/s?
YES/NO – please see consent form

N/A

REFLECTION

**Learnings from case study**
• Top issue is housing, which affects all aspects of physical and mental wellbeing, for both adults and children in the household<br>• Multiple issues with vulnerable families<br>• SPLW feeling helpless to do much about the housing situation
