# Supplementary material for: Practitioner reported barriers to parental engagement with Child Health Hubs in North West London disadvantaged neighbourhoods: a COM-B framework analysis
Source: BMJ Paediatr Open. 2026 Oct 22;10(1):e005272. doi: 10.1136/bmjpo-2026-005272 (PMC13691901; doi:10.1136/bmjpo-2026-005272)
Supplement: Supplementary file 2 [file bmjpo-10-1-s002.pdf]

Supplementary Material 2. Case study data grouped into COM-B domains

Case ID	Capability		Opportunity		Motivation	
	**Physical**	**Psychological**	**Physical**	**Social**	**Reflective**	**Automatic**
CS1	None recorded.	• Mental health challenges: reported severe anxiety and ongoing stress related to relationship breakdown, housing insecurity, financial constraints and childcare.	• Housing insecurity: damp and mould, facing eviction.<br>• Financial constraints: unable to afford rent.<br>• Competing priorities: caring responsibility for three children: Two children aged <4, one with speech and language concern.	• Social isolation: limited partner support, ongoing safeguarding issues with father due to previous episodes of domestic abuse.	None recorded.	None recorded.
CS2	None recorded.	• Mental health challenges: stress and anxiety linked to cultural pressures.	• Financial constraints reported.	• Social isolation: Single mum. Feeling lonely/isolated.	• Past negative experiences/distrust of professionals: rooted in past negative experiences (e.g., non-responsive health visitor), leading to reluctance in disclosing full details for support.	None recorded.

CS3	• Language barrier: mother does not speak English; father speaks English well.	• Mental health challenges: mother experienced perinatal mental health difficulties, multiple miscarriages before having twin, and a stillbirth.	• Housing insecurity: family of seven, overcrowded.<br>• Competing priorities: caring responsibilities for five children: three older boys with autism spectrum disorder (ASD), twin boys <1 years.	None recorded.	None recorded.	None recorded.
CS4	None recorded.	• Parenting confidence & self-efficacy: mother feels she is not useful in supporting her child and places pressure on herself.	None recorded.	• Social Isolation: mother reported feeling isolated and lacking social connections after giving birth; no family nearby apart from partner and in laws; challenges in relationships with in-laws; absence of wider support network.	None recorded.	• Emotional stress: mother described being tearful, emotionally distressed, and struggling to cope.
CS5	• Language barrier noted	• Mental health challenges: single parent caring for three children, struggling with emotional burden of managing multiple responsibilities.<br>• Housing is a major issue affecting mental health.	• Housing insecurity: evicted.<br>• Competing priorities: sole responsibility for three children: aged 2, 7, and 8. The 8-year old has ASD and is still in nappies, the 2-year-old has severe eczema, and all require intensive care.	• Social Isolation: absence of extended family support, parent care managed alone as single parent.<br>• Previously discharged by social worker.	None recorded.	• Emotional stress: parent experiencing heightened stress, fatigue and overwhelmed.

CS6	• Language barrier: family arrived in the UK through relocation from another country. Limited English restricts access to support.	• Service literacy: new to the UK, unfamiliar with available services; required support with universal credit application.	• Housing insecurity: eviction notice received.<br>• Financial constraint: rent arrears/debt.	None recorded.	None recorded.	None recorded.
CS7	None recorded.	• Service literacy: uncertainty about accessing ongoing educational support (homeschooling); lack of confidence navigating systems; difficulties managing practical aspects of caring for twin boys with ASD, including toilet training, restrictive diet, and homeschooling.	• Financial constraint: mother reported not having funds for essential items (e.g., pushchair for twins).<br>• Competing priorities: parents caring for twin boys with ASD alongside caring for elderly grandparents	• Social isolation: responsibility in caring lead to strain and limited social time.	None recorded.	• Emotional stress: ongoing worry and fatigue from high caregiving load.
CS8	• Language barrier noted	• Parenting confidence & self-efficacy: difficulty with breastfeeding, felt low due to challenges and adjusting to motherhood.	None recorded.	• Social isolation: mother reported feeling lonely and keen to make new friends.	• Fear of judgement: reluctance to share issues, still hesitant to disclose needs fully.	• Emotional stress: stress, low mood, tearfulness, and frustration around breastfeeding reduced coping capacity.

CS9	None recorded.	• Mental health challenges: depression, anxiety (both parents), reduced coping capacity.	• Housing insecurity: damp and mould in home, contributed to child's hospitalisation (2 months).<br>• Competing priorities: five children to care for, two with ASD. Overwhelmed and in need for support.	• Social isolation: mother has no extended relationships or friends; difficulty making social connections.	• No perceived benefit engaging shaped by past negative experiences: feeling unsupported during child's hospitalisation.	• Emotional stress: anxiety and low mood, reduced ability to cope and engage consistently.
CS10	None recorded.	• Mental Health challenges: low mood, anxiety about children's health and weight.<br>• Parenting confidence & self-efficacy: concerns about caring for baby and older child with ASD; reported criticism/blame from extended family undermining confidence.	• Financial constraints: struggling with costs, including weight loss support and childcare.	• Family interference/stigma: received criticism/blame from family regarding older child having ASD (negative social environment/discrimination).	None recorded.	None recorded.

CS11	None recorded.	• Mental health challenges: mother struggling with bereavement (loss of her own mother).	• Financial constrain: required help with financial issues and food necessities.<br>• Competing priorities: single mother of three girls (2-year-old has communication needs). Needed to find a nursery for daughter.	• Social isolation: single parent, limited support network.	None recorded.	• Emotional distress: emotional distress from bereavement and divorce, leading to overwhelming feelings and reducing ability to cope with parenting and decision making.
CS12	None recorded.	• Mental health challenges: ongoing mental health struggles has been noted, including appetite changes and low mood.<br>• Parenting Confidence & self-efficacy: mother reported difficulties with self-esteem and confidence, compounded by family interference; expressed desire to get out and about with baby but felt limited by circumstances.<br>• Challenges with breastfeeding and adjusting to motherhood was also observed.	• House insecurity: overcrowded living situation: mother, husband, 3 month-old baby, in-laws, and husband's sibling in one flat; limited mobility and feelings of confinement.	• Family interference: Family member is critical to mother's approach to manage motherhood.	None recorded.	None recorded.

CS13	None recorded.	• Mental health challenges: mother expressed anxiety, particularly around daughter's development and financial insecurity.<br>• Service literacy: limited confidence in navigating services; required support to access nurseries, community resource and complete Universal Credit application.	• Financial constraint: concerns about benefit entitlement and financial security during maternity leave.<br>• Competing priorities: mother currently on maternity leave, expressed worries about leaving baby at nursery when returning to work.	None recorded.	None recorded.	None recorded.
CS14	• Language barrier: recently relocated from abroad; mother does not usually leave the house without her husband due to limited English.	• Mental health challenges: perinatal mental health difficulties; anxiety linked to language barriers, social isolation.<br>• Parenting confidence and self-efficacy: anxiety around childcare and motherhood; fears about baby's health/death.	None recorded.	• Social isolation: lack of established networks in new area; no local friends, no support network.	• Fear of judgement: mother is reluctant to attend community groups.	None recorded.

CS15	None recorded.	• Parenting confidence & self-efficacy: mother and father of newborn. Mother reported difficulties with breastfeeding and latching, which contributed to low confidence and stress around infant care and early motherhood; worries about infant feeding and weight gain created practical challenges for parenting.	None recorded.	None recorded.	None recorded.	None recorded.
CS16	None recorded.	• Mental health challenges: mother has history of depression, currently experiencing stress linked to eldest child’s court case. Anxiety prevents her from leaving the house, and she has a history of overdose attempt.<br>• Dependence on others: Relies on another mother for afternoon school pick-up and on her son to accompany her for dropoffs and shopping.	• Financial constraint reported	• Social isolation: single parent of three children with different fathers. Limited ability to leave house reduces opportunities for social contact and support.	None recorded.	None recorded.

CS17	• Language barrier: English is second language, limiting communication and access to support.<br>• Caregiving capacity (physical health): mother recovering from leukaemia, affecting physical health and ability to manage childcare demands.	• Parental confidence & self-efficacy: mother reported need for reassurance and support in caring for her child, compounded by isolation.	None recorded.	• Social isolation: marriage breakdown; relocated from another country with no close family nearby; father of child involved but working; mother described feeling isolated, lacking parental and local support networks	• Fear of judgement: Mother found social situations very stressful, expressed belief that others were judging her.	None recorded.
CS18	• Language barrier: mother from non-English speaking country with limited English.	• Mental health challenges: past trauma experienced. Ongoing fatigue reported due to childcare during nighttime.<br>• Parenting confidence: mother experienced high anxiety around baby's health (vomiting, overfeeding concerns, constant need for A&E), reflecting low confidence in early parenting.	• Financial constraint: food affordability.	None recorded.	• Fear of judgement (due to past trauma): mother predominantly stays indoors, lacks confidence, and is reluctant to engage in community settings.	None recorded.

CS19	None recorded.	• Mental health challenges: mother has a history of schizophrenia and ongoing perinatal mental health difficulties.<br>• Parenting confidence & self-efficacy: reported lack of confidence as a mother caring for her 8-month-old baby girl. Anxiety about infant care tasks created barriers to confident caregiving. Low confidence led to restrictive behaviours (e.g., preventing the baby from crawling due to fears of injury), limiting the baby's developmental opportunities. Concerns also noted about introducing certain foods (e.g., solid food).	None recorded.	• Social isolation reported.	• Fear of judgement: mother expressed that "no one thinks I can do it", indicating low self-belief and concerns over being judged by others.	• Emotional stress: very anxious and emotionally reactive to minor health concerns; felt overwhelmed and reliant on parents and husband for constant support.

CS20	None recorded.	• Mental health challenges: mother has history of depression, anxiety, eating disorder, and self-harm; no prior therapy despite two historical trauma. Heightened anxiety due to partner's imprisonment.	• Competing priorities: single parent caring for six children, including a 7 month-old infant and a 4-year-old with an Early Help Assessment and Plan (unregulated at school and struggling with toileting). These responsibilities created overwhelming demands, leaving limited time for childcare and engagement. The mother reported constant exhaustion and expressed a need for respite and time to recover.	• Social isolation: single parent; lack of partner support has increased feelings of isolation and stress. Following discharge by social services, mother became further isolated and had reduced access to support.	None recorded.	None recorded.

CS21	None recorded.	• Health literacy: parents lacked knowledge on correct management of their children’s respiratory conditions (3 children with multiple atopic diseases: asthma, wheeze, hay fever, and eczema). Household contained over 20 different medications.	• Housing insecurity: Mould (exacerbate respiratory problems).	None recorded.	None recorded.	None recorded.
