# Supplementary material for: Practitioner reported barriers to parental engagement with Child Health Hubs in North West London disadvantaged neighbourhoods: a COM-B framework analysis
Source: BMJ Paediatr Open. 2026 Oct 22;10(1):e005272. doi: 10.1136/bmjpo-2026-005272 (PMC13691901; doi:10.1136/bmjpo-2026-005272)
Supplement: Supplementary file 3 [file bmjpo-10-1-s003.pdf]

Supplementary Material 3. Enabling factors for parental engagement with integrated services

Case ID	Enabling factors
CS 1	• Multi-disciplinary collaboration: law support, council support, speech and language therapy for her child.<br>• Knowledge of services: Increased awareness of services after signposting.
CS 2	• Community engagement: attendance at group activity improved social support.<br>• Trust Building: sustained engagement with practitioners helped strengthen trust, and mother reported increased confidence in parenting.<br>• Incentive: food voucher .
CS 3	• Readiness/willingness for change: father expressed keenness for professionals to visit the home, demonstrating openness to engagement.<br>• Language support: interpreter support available through Maternity champion, assisting with communication.<br>• Ongoing support is required so feel listened to.
CS 4	• Community engagement: invited to postnatal group and Children's Centre activities.<br>• Ongoing support: regular check-ins helped mother feel listened to, supported, and encouraged to stay engaged.
CS 5	• Ongoing support: continued monitoring and regular check-ins from practitioners provide ongoing encouragement and support.
CS 6	• Community engagement: connected to cultural services. Family referred to toddler/youth groups. Advocacy support (culturally sensitive support):<br>• CHWW liaised with universal credit team on father's behalf.
CS 7	• Multi-disciplinary collaboration: connected to Child and Adolescent Mental Health Services, ADHD centre, Education Department.<br>• Community engagement: attends parent forum, reported it as very helpful.<br>• Incentive: one-off funding secured for pushchair.
CS 8	• Community engagement: joined different community groups, which improved social connections.<br>• Multi-disciplinary collaboration: accessed therapist advice, postnatal support group, and GP medical advice.
CS 9	• Multi-disciplinary collaboration: Talking therapies, Child and Adolescent Mental Health Services. Speech and language therapy, community perinatal support, Home-Start, law support.
CS 10	• Community engagement: accessed postnatal support groups, community groups, and peer connections with other mothers.<br>• Readiness/willingness for change: self-referred to Talking Therapies, attended multiple groups, demonstrating proactive engagement.<br>• Ongoing support: regular check-ins helped sustain trust and engagement.
CS 11	• Peer support: engaged with another mother at Children's Centre with similar needs, mutual support formed.<br>• Willingness for change: sought help for daughter's communication and schooling needs, demonstrating commitment despite challenges.
CS 12	• Multi-disciplinary collaboration: directed to antenatal and other support organisations.<br>• Knowledge of services: improved awareness of available support options.
CS 13	• Multi-disciplinary collaboration: linked with Talking Therapies, Citizens Advice, and Law Centre.
CS 14	• Multi-disciplinary collaboration: referred to language classes (ESOL), children's centre, and community groups.<br>• Access to social opportunities: provided with links to English language learning and parenting support services.
CS 15	• Ongoing support: facilitator provided breastfeeding advice, regular check-ins, and continued monitoring; support addressed both feeding issues and parental wellbeing, strengthening confidence in parenting.

CS 16	• Ongoing support: regular contact with family support worker; on medication for depression.<br>• Community engagement: attend toddler groups, supported by workers.<br>• Incentives: food vouchers for financial assistance.
CS 17	• None recorded.
CS 18	• Ongoing support and trust building: practitioner met mother at the children's centre to reduce barriers to first-time attendance and build confidence. The practitioner accompanied her to group sessions and baby health checks, which helped facilitate her independent participation in future activities.<br>• Incentives: food voucher.
CS 19	• Trust building: ongoing support and reassurance helped build the mother's confidence to reach out for professional help. The practitioner accompanied her to GP appointments and offered support for attending local playgroups or walks to the park with her baby. The team also responded promptly when the mother directly sought help, reinforcing trust and reliability.<br>• Incentives: food bank referral.
CS 20	• Multi-disciplinary collaboration: linked to GP, perinatal mental health team, National Association for Mental Health, moving to Solids workshop, and group play sessions.<br>• Ongoing support: regular contact with services provided reassurance and a safety net.<br>• Incentives: food bank vouchers.
CS 21	• Ongoing support: practitioner spent time rationalising/labelling medicine routines, reinforcing correct use, and educating the family on appropriate medicine use. Family provided positive feedback on the professional's support.<br>• Trust building: practitioner spent extended time with the family, supporting medicine routines.<br>• Incentives: dehumidifier (for mould).
