# Supplementary material for: Practitioner reported barriers to parental engagement with Child Health Hubs in North West London disadvantaged neighbourhoods: a COM-B framework analysis
Source: BMJ Paediatr Open. 2026 Oct 22;10(1):e005272. doi: 10.1136/bmjpo-2026-005272 (PMC13691901; doi:10.1136/bmjpo-2026-005272)
Supplement: Supplementary file 4 [file bmjpo-2026-005272.reviewer_comments.pdf]

PEER REVIEW HISTORY

BMJ Paediatrics Open publishes all reviews undertaken for accepted manuscripts. Reviewers are asked to complete a checklist review form and are provided with free text boxes to elaborate on their assessment. These free text comments are reproduced below.

ARTICLE DETAILS

**TITLE (PROVISIONAL)**	Practitioner reported barriers to parental engagement with Child Health Hubs in North West London disadvantaged neighbourhoods: a COM-B framework analysis
**AUTHORS**	Qin, Jin<br>Blair, Mitch<br>Hargreaves, Dougal<br>Ram, Bina

VERSION 1 – REVIEW

**REVIEWER NAME**	Chong, Lauren
**REVIEWER AFFILIATION**	Sydney Local Health District, Community Paediatrics
**REVIEWER CONFLICT OF INTEREST**	No Conflict of Interest
**AI DISCLOSURE**	No, I have not used AI
**DATE REVIEW RETURNED**	19-Aug-2026

**GENERAL COMMENTS**	Thank you for your submission. This is an important and interesting topic, with a meaningful discussion of barriers and enablers. The paper was easy to read throughout.<br><br>My comments are below:<br>1. The manuscript frames the findings as parents' experiences and barriers to parental engagement, but the primary data are practitioner-generated accounts rather than data collected directly from parents. Practitioners' interpretations of parental behaviour may differ from parents' own accounts, particularly for constructs such as motivation, fear of judgement, trust, confidence and mental health. I suggest that the authors reconsider the framing throughout the manuscript, including the title, aims, results and conclusions, and consistently describe these as practitioner-reported/perceived barriers to parental engagement.<br><br>2. Could you please further clarify your methodology<br>a. How were the 21 cases selected? The paper suggests that these were the only available cases, however is this because they were the only cases where parents engaged sufficiently to record data? If so, this may contribute to some selection bias in the interpretation of the results, and should be discussed as a limitation.<br>b. Please explain the difference in case numbers between the sites eg: Harrow and Brent?<br>c. How was case study data collected? For example, were practitioners recalling conversations retrospectively, was it part of documented notes, or were patient comments recorded verbatim?

**REVIEWER NAME**	Sohn, Jenny
**REVIEWER AFFILIATION**	Sydney Children's Hospital Randwick

**REVIEWER CONFLICT OF INTEREST**	No Conflict of Interest
**AI DISCLOSURE**	No, I have not used AI
**DATE REVIEW RETURNED**	02-Sep-2026

**GENERAL COMMENTS**	1. Further detail on recruitment and data collection methodology. Please provide more detail on the practitioner-generated account approach, including how the case study template was developed and how practitioners were guided in completing it. Please clarify how cases were identified and recruited, who approached potential participants, and whether any eligibility criteria were applied.<br><br>2. Details regarding who completed the case study templates would be more appropriately reported in the methods section rather than the results.<br><br>3. Please clarify which cases were selected for inclusion and the rationale for their selection. It would also be useful to explain the difference in the number of cases included across the three child health hubs.<br><br>4. Consider including an example of a completed case study template as a supplementary file. This would help readers better understand the data collected and how the case studies were structured.<br><br>5. Given that the case studies were practitioner-generated accounts, I suggest making it more explicit throughout the manuscript that the data reflect the clinician/practitioner perspective. This is particularly important given that the stated aim was to explore the barriers parents face in accessing and engaging with integrated child health services. It would be helpful to clarify how the clinician perspective was incorporated into the completion and interpretation of the case study templates, and to acknowledge that the findings represent practitioners' accounts of parents' experiences rather than parents' direct accounts.

**REVIEWER NAME**	Woolfenden, Susan
**REVIEWER AFFILIATION**	The University of Sydney Faculty of Medicine and Health, Community Paediatrics
**REVIEWER CONFLICT OF INTEREST**	No Conflict of Interest
**AI DISCLOSURE**	To transcribe my voice notes directly as I read the paper and then put in the correct grammar and spelling. I then checked and altered the transcribed material. No generative AI was used
**DATE REVIEW RETURNED**	02-Sep-2026

**GENERAL COMMENTS**	Dear Author<br>Thank you for an important paper. I have a number of suggestions for clarification.<br>General comments<br>An important paper. The methods could have more detail, in terms of how the COM-B framework was used to analyse the case study data, how many case studies were involved, and the period of time over which this occurred. In addition to the case studies, some information on how the information from the in-person visits, phone calls, messages and emails was triangulated together would also be

	useful in the methods. I think in the conclusions, firmer recommendations about the kinds of things services could do to engage families would strengthen the messaging.<br><br>Introduction<br>It is well written. I do think it is interesting to comment on behavioural elements and attitudinal elements — that is from the viewpoint of a service system as to what parents should believe, and that is quite different to what a parent might believe is important from a behavioural or attitudinal element. So I think the positionality of the research needs to be declared as this is a providers view in the methods. For example, adherence to appointments and treatment tasks, if these have not been made in collaboration with a parent, may actually not make sense to a parent from a behavioural element and, in fact, may not be appropriate. Some literature on what parents have told us matters to engage with systems eg trauma informed care, trust etc would be good here.<br>I don't think parents only function as critical gatekeepers in accessing and sustaining services, they are your critical partners. It would be good to change the tone around this. I am glad to see the words around co-production and the importance of that, and it is good that you brought it up. I can't see, though, how the issue of parental input is actually answered by this paper, as there does not seem to be a co-production element, or how the COM-B framework relates to the co-production. It would be good to make that clearer. Are you using the COM-B framework as a first part of co-production, to understand why families might engage with services, and is the next step then to work with families around this?<br>You identify parental perceptions of integrated health services as a clear gap. However, my understanding is that the perceived barriers are from case studies put together by service providers. So was there any parent oversight of the findings and interpretation in terms of the COM-B framework? This would be important to address.<br><br>Methods<br>There could be much more on how the case studies were done. It is good to see that this is in the supplementary material, but I think there needs to be a little for those uninitiated around how a case study is a rigorous way of addressing this question; how the case studies were decided upon; how, if you like, the settings were decided upon; and how the recruitment of these case studies was decided upon. Was open inductive thematic analysis used to originally look at the qualitative themes? Could you please reference the qualitative methods literature on topics such as thematic saturation. It is good to see the patient and public involvement illustrated in this study; however, it would be important to see how that was used in the analysis of the case studies, if at all, as that is not clear.<br><br>Results<br>The results are interesting and rich. It would be good to have an example of a case study (beyond the table) for the reader to understand the interpretation by the clinician of the mother's story. My main criticism is that the data seems quite deficit focussed. All

	families have strengths that Hubs build on but I can't see these highlighted eg family strong in their culture and community<br><br>Discussion and limitations<br>It is good to see the limitation of parents' experience being generated by practitioners noted. I think we should also speak to the power differential that this brings<br>in when we are talking about engagement with priority populations, and what future research should do around that I would like to see some reflection on the barriers that were intrinsic to the hubs themselves and the way the service system might have worked eg the way staff may speak to a parent, siloed ways of working, to balance the parental driven issues.

VERSION 1 – AUTHOR RESPONSE

**Comment**	**Response**	**Amendment (if applicable)**
**Reviewer 1: Dr. Lauren Chong, Sydney Local Health District**		
Thank you for your submission. This is an important and interesting topic, with a meaningful discussion of barriers and enablers. The paper was easy to read throughout.	We thank Dr Laura Chong for this feedback.	
1. The manuscript frames the findings as parents' experiences and barriers to parental engagement, but the primary data are practitioner-generated accounts rather than data collected directly from parents. Practitioners' interpretations of parental behaviour may differ from parents' own accounts, particularly for constructs such as motivation, fear of judgement, trust, confidence and mental health. I suggest that the authors reconsider the framing throughout the manuscript, including the title, aims, results and conclusions, and consistently describe these as practitioner-reported/perceived barriers to parental engagement.	We have changed the framing throughout the paper to better reflect that the work presented is that of practitioner-reported/perceived barriers to parental engagement, including edits to the manuscript title.	**Page 1, lines 1-3**<br><br>*"Practitioner reported barriers to parental engagement with Child Health Hubs in North West London disadvantaged neighbourhoods: a COM-B framework analysis."*
2. Could you please further clarify your methodology<br>(a) How were the 21 cases selected? The paper suggests that these were the only available cases,	(a) The 21 case studies were those which fit the criteria of a family with a child aged 0-5 years and permission from the family for their data to be	**(a) Page 5 lines 155-156**

however is this because they were the only cases where parents engaged sufficiently to record data? If so, this may contribute to some selection bias in the interpretation of the results, and should be discussed as a limitation.<br>(b) Please explain the difference in case numbers between the sites eg: Harrow and Brent?<br>(c) How was case study data collected? For example, were practitioners recalling conversations retrospectively, was it part of documented notes, or were patient comments recorded verbatim?	shared with the evaluation team. Unfortunately we were not provided with detail from the sites of how these cases were selected. We have added these points to our methods and to the limitations to address potential selection bias.<br>(b) We have now included an explanation of the differences in number of cases provided by the sites.<br>(c) We have now added information to the methods to clarify how study data was collected.	*"Data of interest were families with children aged 0–5 years."*<br><br>**Page 6, lines 194-196**<br><br>*"Only case studies where practitioners had obtained permission from families were shared with the evaluation team."*<br><br>**Page 14, lines 431-432**<br><br>*"We did not have access to detail describing how HLPCTs identified families to include for the case studies and how these were selected to be shared with the evaluation team."*<br><br>**(b) Page 7, lines 209-211**<br>*"Differences in the number of case studies provided by each site, specifically the limited number from Ealing and Brent compared with Harrow, reflected delays to pilot start dates and limited resources[21] to complete case study templates."*<br><br>**(c) Page 5, lines 154-156**<br>*"These were compiled by early years practitioners working with each early years pilot site. Data of interest were families with children aged 0–5 years."*<br><br>**Page 5, lines 160-168**<br><br>*"Case study data were captured using a template*

		(Supplementary Material 1) designed by the Optivita (Harrow) Hyper Local Preventative Care Team (HLPCT). The template included specific questions and required minimal guidance to complete. HLPCTs at each pilot site identified families to include as case studies. The data comprised practitioner-generated accounts of families' experiences, challenges, support received, and perceptions of Child Health Hub services. The templates were completed retrospectively; practitioners referred to their recorded notes during patient contact (in-person home visits/meetings/events, phone calls, emails, text messages). The final section of the template requested practitioners' own reflections and learning from each case."
**Reviewer 2: Dr. Jenny Sohn, Sydney Children's Hospital Randwick, University of New South Wales**		
3. Further detail on recruitment and data collection methodology. Please provide more detail on the practitioner-generated account approach, including how the case study template was developed and how practitioners were guided in completing it. Please clarify how cases were identified and recruited, who approached potential participants, and whether any eligibility criteria were applied.	Please see response to #2(c) where we have added further detail of how the case study template was developed, how data was collected, addressed selection of case studies, and how practitioners were guided in completing the template.	
4. Details regarding who completed the case study templates would be more appropriately reported in the methods section rather than the results.	We have moved the detail of who completed the case study templates to the methods sections.	**Page 5-6, lines 168-171**<br><br>*"Practitioners' who compiled the case studies were community workers (Social Prescribing Link Workers (SPLWs),*

		Community Health and Wellbeing Workers (CHWWs), Family Link Workers (FLWs)), respiratory clinic practitioners, and those facilitating group-based activities (e.g., Children's Centres)."
5. Please clarify which cases were selected for inclusion and the rationale for their selection. It would also be useful to explain the difference in the number of cases included across the three child health hubs.	Please refer to response #2 (a) and (b) where we have addressed selection and inclusion of participants, and added an explanation of the differences in the number of cases included across the three child health hubs.	
6. including an example of a completed case study template as a supplementary file. This would help readers better understand the data collected and how the case studies were structured.	Supplementary Material 1 has now been adapted to include a case study.	
7. Given that the case studies were practitioner-generated accounts, I suggest making it more explicit throughout the manuscript that the data reflect the clinician/practitioner perspective. This is particularly important given that the stated aim was to explore the barriers parents face in accessing and engaging with integrated child health services. It would be helpful to clarify how the clinician perspective was incorporated into the completion and interpretation of the case study templates, and to acknowledge that the findings represent practitioners' accounts of parents' experiences rather than parents' direct accounts.	Please see response to #1 in which we have changed the framing throughout the paper to better reflect that the work presented is that of practitioner-reported/perceived barriers to parental engagement. Through being explicit that the case studies were practitioner-generated accounts, there is better clarification throughout the manuscript that the interpretation is based on practitioner perspectives of parents' experiences and findings are not directly parents' accounts.	
**Reviewer 3: Prof. Susan Woolfenden, The University of Sydney Faculty of Medicine and Health**		
Thank you for an important paper. I have a number of suggestions for clarification.	We thank Professor Susan Woolfenden for this feedback and have addressed all the comments raised.	
8. General comments<br>An important paper.<br>(a) The methods could have more detail, in terms of how the COM-B framework was used to analyse the case	(a) We have added more detail about how the COM-B framework was used to analyse the case study data.	**(a) Page 6, lines 175-177**

study data, how many case studies were involved, and the period of time over which this occurred.<br>(b) In addition to the case studies, some information on how the information from the in-person visits, phone calls, messages and emails was triangulated together would also be useful in the methods.<br>(c) I think in the conclusions, firmer recommendations about the kinds of things services could do to engage families would strengthen the messaging.	The number of case studies involved had been provided in the Results (page 7, line 207), and we have now added clarification that we did not have information on how many case study data were collected in total from which 21 were shared with us. The Methods had described the period of time when these occurred which we have revised. We have added detail to our limitations to explain that we were not provided with information of how many case studies were completed and how those shared with the evaluation team were selected.<br>(b) We did not directly triangulate the information from the different types of contact practitioners had with families. Practitioners referred to their recorded notes to provide a summary of information. We therefore did not capture information recorded by type of contact per family. We have added detail to our methods for clarification.<br>(c) We have revised our conclusion to suggest firmer recommendations.	*"The COM-B framework[23] was integrated deductively within an interpretivist approach[24] to provide a systematic means of categorising the behavioural determinants of practitioner accounts of parental engagement."*<br><br>**Page 6, lines 194-196**<br>*"Only case studies where practitioners had obtained permission from families were shared with the evaluation team."*<br><br>**Page 5, lines 153-154**<br>*"…were collected for the NWL Early Years GP Child Health Hubs Pilots evaluation between September 2024 and January 2025."*<br><br>**Page 14, lines 431-432**<br>*"We did not have access to detail describing how HLPCTs identified families to include for the case studies and how these were selected for sharing with the evaluation team."*<br><br>**(b) Page 5, lines 165-168**<br>*"The templates were completed retrospectively; practitioners referred to their recorded notes during patient contact (in-person home visits/meetings/events, phone calls, emails, text*

		messages). The final section of the template requested practitioners' own reflections and learning from each case."<br><br>**(c) Pages 16-17, lines 507-517**<br>*"As reported by practitioners, families living in disadvantaged areas of NWL face multiple, intersecting barriers to access integrated child health services. To achieve equitable access, Child Health Hubs should provide integrated support addressing health and social needs together, rather than through fragmented interventions. Proactive approaches to reducing barriers could include flexible and accessible provision, community based outreach, sustained relationships with families, and building trust with local organisations to ensure services are accessible, acceptable, and responsive. However, longer-term evaluation is needed to understand how parental engagement evolves over time, particularly how participation can be sustained among families experiencing adversity."*
9. Introduction<br>It is well written.<br>(a) I do think it is interesting to comment on behavioural elements and attitudinal elements — that is from the viewpoint of a service system as to what parents should believe, and that is	(a) Please see response to #1 where we have made changes throughout the manuscript to better reflect that the work presented is that of practitioner-reported/perceived barriers (and not directly reported by parents).	**(a) Page 4, lines 116-119**<br>*"Engagement with services has been shown to be facilitated by parents' perceived benefits, content*

quite different to what a parent might believe is important from a behavioural or attitudinal element. So I think the positionality of the research needs to be declared as this is a providers view in the methods. For example, adherence to appointments and treatment tasks, if these have not been made in collaboration with a parent, may actually not make sense to a parent from a behavioural element and, in fact, may not be appropriate. Some literature on what parents have told us matters to engage with systems eg trauma informed care, trust etc would be good here.<br>(b) I don't think parents only function as critical gatekeepers in accessing and sustaining services, they are your critical partners. It would be good to change the tone around this.<br>(c) I am glad to see the words around co-production and the importance of that, and it is good that you brought it up. I can't see, though, how the issue of parental input is actually answered by this paper, as there does not seem to be a co-production element, or how the COM-B framework relates to the co-production. It would be good to make that clearer. Are you using the COM-B framework as a first part of co-production, to understand why families might engage with services, and is the next step then to work with families around this?<br>(d) You identify parental perceptions of integrated health services as a clear gap. However, my understanding is that the perceived barriers are from case studies put together by service providers. So was there any parent oversight of the findings and interpretation in terms of the	In addition, we have added a sentence and referenced literature on lines 83 and 88: Jukes LM, et al., Barriers and Facilitators…*Child Psychiatry and Human Development*, 2022 and Zhang H, et al., Barriers and Facilitators to Sustained Engagement… *Research on Social Work Practice*, 2026, which addresses parents perspectives of engaging with healthcare systems<br>(b) We have amended this sentence to change the tone around parents being critical gatekeeps to also being critical partners.<br>(c) We have re-worded the aim of the study in both the abstract and introduction to clarify that the purpose of this paper was to apply the COM-B framework to identify why parents may not engage with services.<br>(d) There was no parent oversight of the findings and interpretations in terms of the COM-B framework. We have now added this as a limitation of our study.	*that is practical and culturally appropriate, flexible delivery, supportive environments, and responsive facilitator practices.[9]"*<br><br>**(b) Page 4, lines 114-116**<br><br>*"…parents function as critical partners and gatekeepers in access and sustained engagement with services;…"*<br><br>**(c) Page 2, lines 20-21**<br><br>*"This study explored practitioner-reported barriers parents face in accessing and engaging with integrated child health services."*<br><br>**Page 5, lines 145-148**<br><br>*"The aim of this study is to use practitioner-reported case-study data, collected as part of the NWL Early Years GP Child Health Hubs Pilots evaluation, [21]to understand the perceived barriers that underserved communities experience when engaging with Child Health Hubs in NWL."*<br><br>**(d) Page 14, lines 433-434**<br><br>*"…generated by practitioners', with no parental oversight into the interpretation of the findings."*

COM-B framework? This would be important to address.		
10. Methods<br>(a) There could be much more on how the case studies were done. It is good to see that this is in the supplementary material, but I think there needs to be a little for those uninitiated around how a case study is a rigorous way of addressing this question; how the case studies were decided upon; how, if you like, the settings were decided upon; and how the recruitment of these case studies was decided upon.<br>(b) Was open inductive thematic analysis used to originally look at the qualitative themes? Could you please reference the qualitative methods literature on topics such as thematic saturation.<br>(c) It is good to see the patient and public involvement illustrated in this study; however, it would be important to see how that was used in the analysis of the case studies, if at all, as that is not clear.	(a) Please see response to #2 where we have added more detail around how the case studies were done.<br>(b) We used a deductive approach to apply the COM-B domains to the data (see response to #8), but the analysis remained open to inductive themes. We have added detail to clarify this and added the following reference for thematic saturation: doi.org/10.1371/journal.pone.0232076<br>(c) The information provided by practitioners in the case study template included a summary of all contact they had with the family, which may have included contact during public events. We have now added clarification.	**(b) Page 6, lines 177-181**<br>*"Inductive approaches were used to identify determinants to ensure the analysis remained responsive to factors emerging from practitioners' accounts that were not anticipated by the COM-B framework.*[23]<br><br>**(c) Pages 6-7, lines 200-202**<br>*"Contact with families during these events were included, if applicable, in the practitioner-generated case study reports."*
11. Results<br>(a) The results are interesting and rich. It would be good to have an example of a case study (beyond the table) for the reader to understand the interpretation by the clinician of the mother's story.<br>(b) My main criticism is that the data seems quite deficit focussed. All families have strengths that Hubs build on but I can't see these highlighted eg family strong in their culture and community	(a) We have amended our Supplementary Material 1 to provide an example case study. Please see response #6.<br>(b) The aim of this study was to use practitioner-generated case study data to identify parental barriers of engaging with Child Health Hubs, hence the deficit focus. We had included a short paragraph on enablers (page 14, lines 381-385), but this was not the focus of our study.	
12. Discussion and limitations<br>It is good to see the limitation of parents' experience being generated by practitioners noted. I think we should also speak to the power differential	We have added a paragraph to our Discussion to address how the barriers may have been intrinsic to the hubs and	**Page 16, lines 486-500**<br><br>*"Our findings may also suggest a need to move beyond accounts of*

that this brings in when we are talking about engagement with priority populations, and what future research should do around that I would like to see some reflection on the barriers that were intrinsic to the hubs themselves and the way the service system might have worked eg the way staff may speak to a parent, siloed ways of working, to balance the parental driven issues.	the way the service system might have worked.	*engagement that locate barriers primarily within parents or their circumstances. Although understanding parental experiences and priorities may directly influence their engagement with Chid Health Hubs, their experiences may also be influenced by the healthcare structures and services with which they interact. Applying the COM-B framework provides an improved understanding of these influences, particularly through the domain of opportunity, which includes the social and environmental conditions that may enable or constrain behaviour, and may be created or reinforced through service structures, professional practices and power imbalances. For example, practitioners' communication with parents, assumptions about parental needs, and fragmented services may influence parents' social and physical opportunities to engage, while power imbalances may influence what is understood as meaningful engagement. Future research should consider COM-B alongside a more critical examination of the service context. This would help to ensure that lack of engagement is not placed predominantly on parents, and instead recognise engagement as a relational process shaped by both parental circumstances and the*

		systems in which services are delivered."
